# Lu Tong Ke Li protects neurons from injury by regulating inflammation in rats with brain trauma

**DOI:** 10.1002/ibra.12029

**Published:** 2022-03-11

**Authors:** Jie Chen, Ting‐Ting Li, Ting‐Bao Chen, Rui‐Ze Niu, Ji‐Lin Chen, Yong Chen, Jin Huang

**Affiliations:** ^1^ Animal Zoology Department, Institute of Neuroscience Kunming Medical University Kunming China; ^2^ Department of Anesthesiology Southwest Medical University Luzhou Sichuan China; ^3^ Department of Anesthesiology, Institute of Neurological Disease, West China Hospital Sichuan University Chengdu China

**Keywords:** Chinese medicine, Inflammation genes, Lu Tong Ke Li, Neuroprotection, Traumatic brain injury

## Abstract

Currently, there is no effective therapy for traumatic brain injury (TBI). Therefore, this study was conducted to determine the protective effect of Lu Tong Ke Li (LTKL), a Chinese medicine, for TBI in experimental animals. The TBI rat model was induced using the modified Feeney's protocol. The rats were divided into four groups: Sham group, Control group, LTKL  lower‐dose group (LTL, 2 g/kg/day, p.o.), and LTKL higher‐dose group (LTH, 4 g/kg/day, p.o.). The Neurological Severity Score (NSS) was used to examine neurological function. Magnetic resonance imaging was performed to check the brain tissue lesions in rats. Cell apoptosis in the damaged area was evaluated using the Terminal deoxynucleotidyl transferase deoxy‐UTP‐nick end labeling assay. Reverse‐transcription polymerase chain reaction was used to investigate the expression of inflammatory cytokines, including tumor necrosis factor‐α (TNF‐α), interleukin 1β (IL‐1β), and interleukin 10 (IL‐10). The TBI rat model was successfully constructed. Neurological function was enhanced at 14, 21, and 28 days post TBI in the LTH groups, indicated by gradually decreased NSS scores. Administration of LTH led to fewer brain defects in the damaged area, and the number of apoptosis cells in the brain injury area markedly decreased. LTKL treatment led to upregulation of IL‐10 expression and downregulation of TNF‐α and IL‐1β expressions at the molecular level. LTKL can improve the neurobehavior of TBI. The neuroprotective effect was probably related to regulation of inflammation cytokines. Our results provide crucial evidence of the potentially useful application of LTKL in the therapy of TBI in clinic practice in the future.

## Graphical abstract

Lu Tong Ke Li (LTKL) can improve the neurobehavior of traumatic brain injury (TBI). The neuroprotective effect was probably related to regulation of inflammation cytokines, including downregulation ofTNF‐α and IL‐1β, and upregulation of interleukin 10 (IL‐10). Our results provide crucial evidence for the potentially useful application of LTKL in the therapy of TBI in clinic practice in the future.

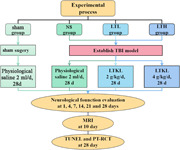

## 1 INTRODUCTION

Traumatic brain injury (TBI) is caused by a sudden external force inflicted on the brain, followed by neurological deficits, which is a major challenge for health care.^1^ The incidences of TBI surpass 1.7 million each year in America.^2^ In China, there are more than 1 million TBI patients every year.^3^ It is an urgent need for effective therapy for TBI patients. Brain trauma can be classified into primary and secondary injury. Primary injury is a direct result of external forces and occurs at the time of the injury, while secondary injury involves a wide variety of processes, including calcium overload, oxidative stress, inflammatory reaction, and mitochondrial dysfunction.^4^, ^5^ These processes may promote nerve cell deterioration and apoptosis, and affect the prognosis of the trauma patients.^6^ Alleviating the injury of TBI patients by reducing the degeneration and apoptosis of nerve cells was one of the treatment methods of TBI.

Inflammation plays an important role in the pathophysiological process of secondary injury.^7^ After brain injury, numerous cellular factors are released within a few minutes, which regulate a series of inflammatory reactions, including the secretion of inflammatory factors, invasion of inflammatory cells, and expression of adhesion molecules, which cause further brain damage.^8^, ^9^ Interleukin 1β (IL‐1β), a pro‐inflammatory cytokine, is an important member of the IL‐1 family.^10^ Under physiological conditions, the substance of IL‐1β was in a low level.^11^ The expression of IL‐1β messenger RNA (mRNA) increases rapidly following brain injury, which has a regulatory effect on inflammation and immune response.^12^ Tumor necrosis factor‐α (TNF‐α), a multifunctional pro‐inflammatory cytokine, was released by microglia and astrocytes.^13^ TNF‐α exerts a neurotoxic effect, and increased expression of TNF‐α causes nerve cell swelling and necrosis.^14^ Interleukin 10 (IL‐10) was an anti‐inflammatory factor, and its major function was to inhibit the production of IL‐1β and TNF‐α.^15^ After brain injury, local glial cells and lymphocytes secrete IL‐10, which can decrease the antigen presentation of microglia cells, prevent the adhesion and invasion of inflammatory cells, and reduce the expression of inflammatory cytokines to ameliorate secondary brain injury.^16^, ^17^

Traditional Chinese Medicine (TCM), with its comprehensive medicinal practices and in use in clinical practice for over 2500 years, is worth exploring as a means of treatment of TBI, and thus, we hope that it is useful for treatment of TBI worldwide.^18^ The validated medical utility of Chinese medicine makes it a reasonable alternative medical approach for TBI therapy.^19^ Previously, a compound Chinese medicine called Lu Tong Ke Li (LTKL) developed by the southwest medical university. It was used to alleviate the symptoms of patients with migraine in the clinic. LTKL was composed of Notopterygium root, Ligusticum, Schizonepeta, Ligusticum, wallichii, Scorpion, Fructus viticis, Pueraria, and so forth, prepared and supplied. The specification is 10 g/bag, and the production batch number is 20161009. By Western Blot and immunohistochemistry, it was found that LTKL could reduce the expression of IL‐1β, TNF‐α, and cyclooxygenase‐2 (COX‐2) proteins in the brain stem of migraine model rats. Therefore, this study was designed to explore the protective mechanism of LTKL against acute inflammatory responses and the cellular apoptosis gene to gain an understanding of the possible role of LTKL as an effective drug for TBI therapy.

## 2 MATERIALS AND METHODS

### 2.1 Animals and maintenance

**Figure 1:**
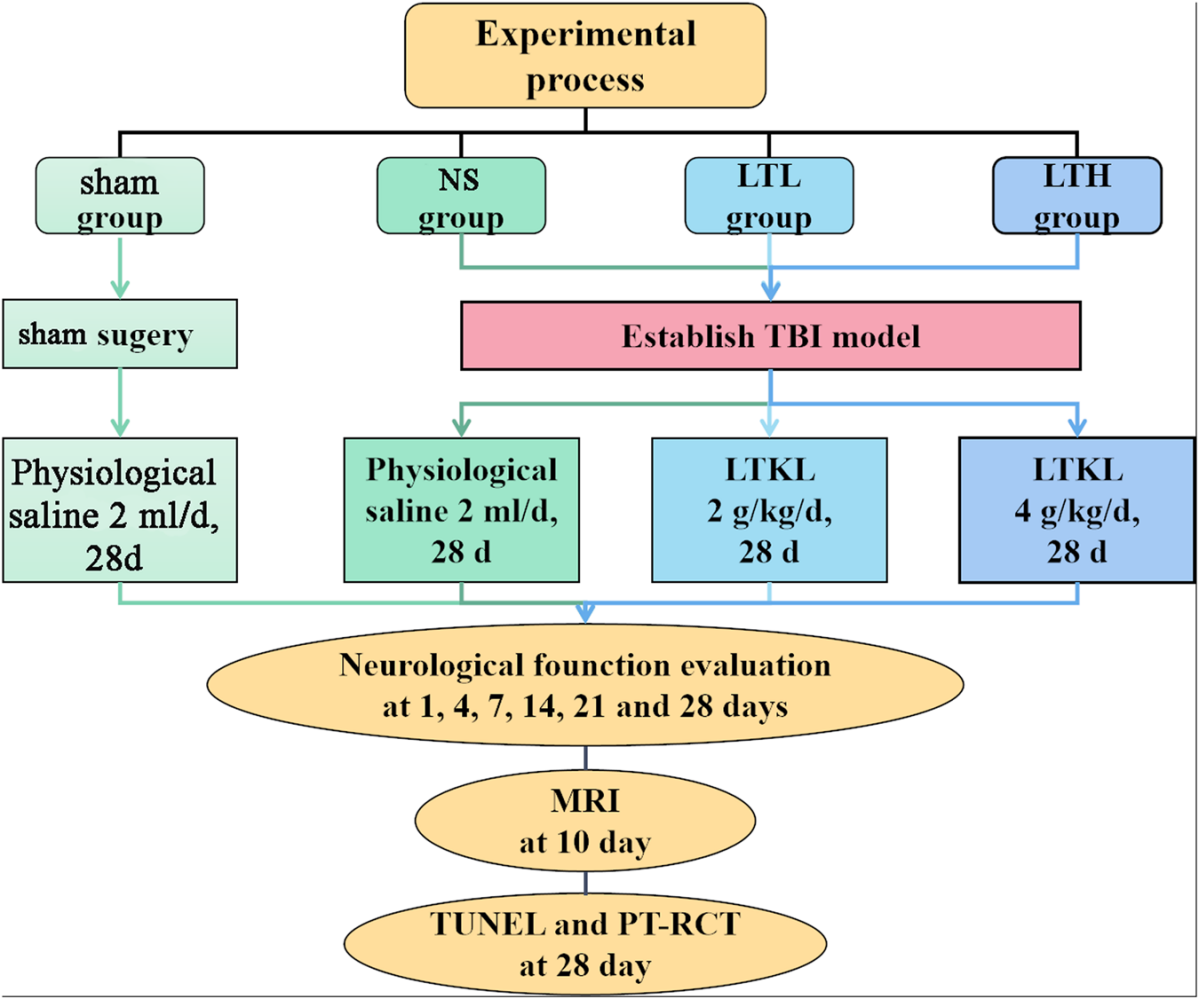
Experimental flowchart [Color figure can be viewed at wileyonlinelibrary.com]

Male adult Sprague–Dawley rats (200–240 g) were obtained from the Laboratory Animal Center of Kunming Medical University. The protocols of the experiment were authorized by the Institutional Medical Experimental Animal Care Committee of Kunming Medical University, China, on October 20, 2020 (No., 2021‐759). They were randomly distributed into the sham group, the control group (NS), the LTKL lower‐dose group (LTL, 2 g/kg/day, p.o.), and the LTKL higher‐dose group (LTH, 4 g/kg/day, p.o.) (Figure 1). Animals were housed in 12 h light/h dark cycle conditions, with food and water available ad libitum (*n* = 10 each group). All experimental protocols conformed to the Guide for the Care and Use of Laboratory Animals formulated by the National Institute of Health (NIH).

### 2.2 TBI model and treatment

The TBI model was developed in accordance with the modified Feeney's method.^20^ The rats were anesthetized with 3% pentobarbital sodium (30 mg/kg, intraperitoneal injection) and placed onto a stereotactic platform. The rats were shaved and sterilized, and then, a 1.5 cm midline scalp incision was made to expose the right parietal bone. A 0.5 × 0.5 cm skull window was created using a dentist's micro‐drill (0.2 cm behind the coronal suture and 0.3 cm lateral to the midline). Then, a 50 g weight was dropped from a height of 25 cm along a vertical gavelock directly onto the exposed lobe. The sham‐operated rats were subjected to the same process, except for impaction. Then, the scalp was closed. According to the dose conversion coefficient table of the animal and human, we set the required dose of the experimental rats in each group as follows: 4 g/kg/day in the LTH group of LTKL and 2 g/kg/day in the LTL group of LTKL. The concentration of the gavage drugs in the higher‐ and lower‐dose groups of rats was calculated according to the standard volume of 2 ml, and the NS group and the sham group were administered 2 ml of normal saline. Rats were administered the corresponding drugs in all groups for 28 consecutive days, and the frequency was once a day in the morning (Figure 1).

### 2.3 Neurological function evaluation

The degree of a neurological defect was evaluated according to NSS by a professional who was blinded to the experimental design.^21^ Assessments using NSS are based on motor, balance, sensory, and reflex responses. The total score is 18, and the normal score is 0. A higher score is indicative of more severe damage (Table 1). The neurobehavioral tests were performed at 1, 4, 7, 14, 21, and 28 days postoperation.

**Table 1:** Neurological Severity Score

Neurological Severity Score		
Evaluation item	Rat behaviors	Score
Lifts tail, about 1 M off the ground (normal 0 points, max. 3 points)	The limbs are extended, the head deviates from the midline by no more than 10 degrees, and the front and back limbs are normal	0
	The head deviated more than 10° from the midline	1
	Fore buckling	1
	Hind legs rigidity	1
Motor function (normal 0, max. 3)	Walk normally	0
	Cannot walk in a straight line	1
	Turn to the paralyzed side, and record left or right deviation	2
	Unable to walk	3
Crossbeam test (normal 0 points, max. 6 points)	Stands steadily on the beam	0
	Standing on the beam, rocking from side to side, but not sliding down	1
	Standing on a beam, one limb slipped, but the rat did not fall off	2
	Standing on the beam, two limbs slipped, but the rat did not fall off	3
	After standing on the beam for 40 s, the rat fell off the beam	4
	Drops off the beam after 20 s	5
	Drops off the beam within 20 s	6
Sensory function (normal 0, max. 2)	Pain and temperature sensation	1
	Proprioception	2
Reflex activity (normal 0, max. 4)	Auricle reflex	1
	Corneal reflex	1
	Panic reflection	1
	Myodystony	1

*Note*: Each score can be superimposed.

### 2.4 Magnetic resonance imaging (MRI)

MRI was used to detect the morphological features of TBI at 10 days postoperation. A Bruker 7.0‐T scanner was used to acquire MRI. Rats were briefly anesthetized with 3% isoflurane using an anesthesia machine for small animals. During anesthesia, the rats' heart rate and respiration were continuously measured to maintain a stable depth of anesthesia. Each rat was fixed in a prostrate position and covered with a small blanket to maintain body temperature. The head of the rat was placed in the center of the coil. T2 images were gathered with a spin echo (SE) sequence, TR/TE = 2500/80 ms, field of view (FOV) = 32 × 32 mm, matrix = 384 × 384, layer thickness = 0.1 cm. The lesion part was analyzed using the ITK‐SNAP 3.6 method (www.itksnap.org).^22^

### 2.5 Terminal deoxynucleotidyl transferase deoxy‐UTP‐nick end labeling (TUNEL) assay

After feeding for 28 days, all the rats were anesthetized with gas and fixed on an anatomical table in the supine position. After the tissue layer was separated and the heart was exposed, 0.9% normal saline was injected into the aorta for perfusion. Immediately after perfusion, the skull was cut open, the whole brain was removed, and one part was frozen and one part was fixed with paraformaldehyde. The frozen samples were stored in an ultra‐low‐temperature refrigerator at −80°C, and the fixed tissues were placed in a 4% paraformaldehyde solution for fixation. Brain tissue was collected, sectioned, and rinsed with phosphate‐buffered saline (PBS, 0.01 mol/l, PH7.4, three times, 5 min each). Then, the tissue was treated with 3% hydrogen peroxide and 0.1% Triton‐X 100/0.1% sodium citrate for 15 min at room temperature. Then, slides were washed with PBS (0.01 mol/l, PH 7.4, three times, 5 min each) and incubated with the TUNEL reaction mixture (Roche Molecular Biochemicals) for 18 h at 4°C. Finally, the slides were washed with PBS using the previous methods and nuclei were redyed with 4′, 6‐diamidino‐2‐phenylindole (DAPI).

### 2.6 Reverse‐transcription polymerase chain reaction (RT‐PCR)

Total RNA was extracted using TRIzol reagent (Invitrogen). Reverse transcription to complementary DNA (cDNA) was performed according to the protocol of the Revert Aid First Strand cDNA Synthesis Kit (Thermo Fisher Scientific). RT‐PCR was performed to determine the degree of inflammation and the number of apoptotic cytokine genes (Table 2). The reaction of amplification was performed in a DNA thermal cycler (ABI 7300) and the fluorescent signal was checked when it reached the threshold. β‐actin, the endogenous control gene, was used to determine the normalized ΔCt value of each sample. According to the standard instruction, proceed the following steps in sequence: one cycle (94°C, 5 min), 35 cycles (94°C, 1 min), and annealing (72°C, 1 min). The relative expression of the targeted gene was determined using the $2^{‐\Delta \Delta C_{t}}$ method.

**Table 2:** Forward and reverse sequences for qPCR

Gene	Forward sequences	Reverse sequences	Annealing temperature
TNF‐α	5′‐GCGTGTTCATCCGTTCTCTA‐3′	5′‐GCGTGTTCATCCGTTCTCTA‐3′	60°C
IL‐1β	5′‐GAGCTGAAAGCTCTCCACCT‐3′	5′‐TTCCATCTTCTTCTTTGGGT‐3′	60°C
IL‐10	5′‐CAGAAATCAAGGAGCATTTG‐3′	5′‐CTGCTCCACTGCCTTGCTTT‐3′	50°C
β‐actin	5′ ‐ GTAAAGACCTCTATGCCAACA‐3′	5′‐GGACTCATCGTACTCCTGCT‐3′	52.5°C

Abbreviations: IL‐1β, interleukin 1β; IL‐10, interleukin 10; qPCR, quantitative polymerase chain reaction; TNF‐α, tumor necrosis factor‐α.

### 2.7 Qualification and statistical analysis

In this study, SPSS 20.0 software (SPSS Inc.) was used to analyze the data, and one‐way analysis of variance was used to determine the statistical differences among groups. All results are shown as mean ± standard deviation (SD). *p* < 0.05 was considered to indicate a statistically significant difference.

## 3 RESULTS

### 3.1 LTKL improves rats' neurobehavior following TBI

**Figure 2:**
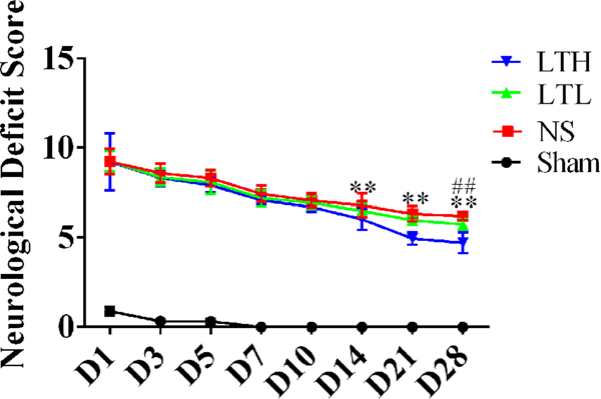
LTKL improved neurological function in rats with traumatic brain injury. Neurological function was determined using the NSS score at 1, 4, 7, 14, 21, and 28 days postoperation. The result is shown as means ± SD. LTH, LTKL higher‐dose; LTKL, Lu Tong Ke Li; NSS, Neurological Severity Score. ^**^The difference between the NS group and the LTH group was statistically significant, *p* < 0.01. ^##^The difference between the LTL group and the LTH group was statistically significant, *p* < 0.01 [Color figure can be viewed at wileyonlinelibrary.com]

At 1, 3, 5, 7, 10, 14, 21, and 28 days postoperatively, we calculated an NSS score for every rat. At each time point, the score from the TBI rats was obviously higher than that of the sham group. However, compared with the NS group, the scores in the LTKL‐treated group were all lower, while in the LTH group, the scores were significantly lower at 14, 21, and 28 days (*p* < 0.05). Scores in the LTH group were lower than those in the LTL group, especially at 28 days. The results show that the effect of LTKL was concentration‐dependent, so high‐dose LTKL was administered to animals with TBI in the experiment (Figure 2). Since the effect was more obvious in the high‐dose group, the differences between the sham group and the LTH group were only determined in the subsequent MRI TUNEL comparison.

### 3.2 MRI

**Figure 3:**
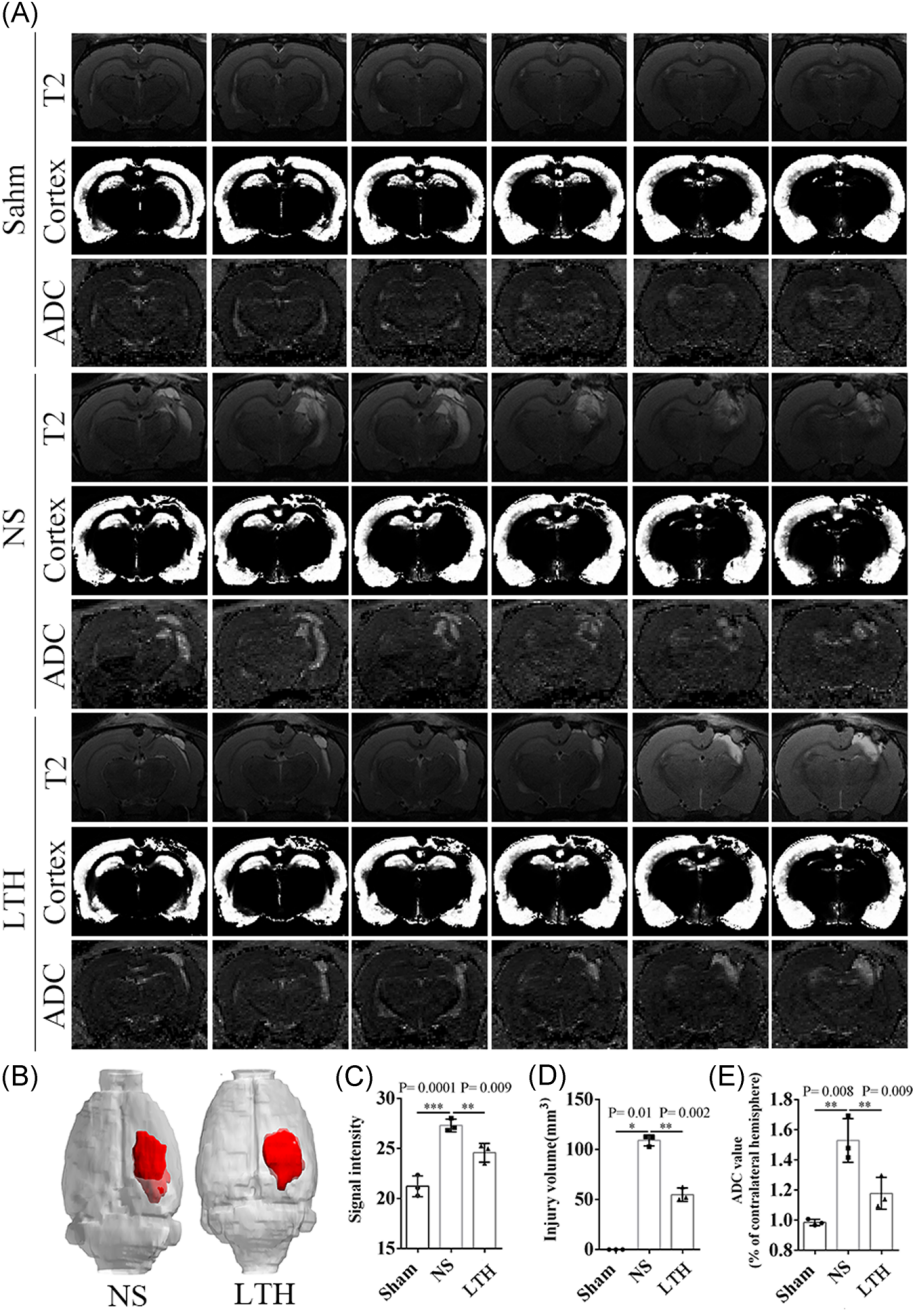
MRI Images of rats with TBI. (A) T2‐weighted imaging and diffusion‐weighted imaging of TBI rats on the 10th day postoperation. (B) Three‐dimensional views of lesion volume. (C) Hemorrhagic edema signals in areas of brain tissue injury were detected by MRI. (D) Volume of injury was determined by MRI. (E) Diffusion‐weighted imaging of the coronal plane of the brain in rats with brain trauma detected by MRI (*n* = 3 each group). Results are shown as mean ± SD. MRI, magnetic resonance imaging; TBI, traumatic brain injury. *p < 0.05, **p < 0.01 [Color figure can be viewed at wileyonlinelibrary.com]

T2‐weighted imaging of the coronal plane of the brain showed significant high‐signal hemorrhage and edema in the injured side of rats. Compared with the sham group, the rats of the NS group showed significantly increased signal and hemorrhage and edema in the damaged side of the brain.  In the LTH group, the signal strength at the injured side was obviously reduced. In terms of morphology, the rats in the NS group had a local defect on the damaged side cerebral cortex, compared with the sham group. However, the rats administered higher‐dose LTKL showed partial recovery of the damaged side cerebral cortex defect with continuous improvement compared with the NS group. Diffusion‐weighted imaging of the coronal plane of the brain showed that compared with the sham group, the ratio of the apparent dispersion coefficient between the damaged lateral brain tissue and the contralateral cerebral tissue increased obviously, and the ratio of diffusion coefficient between the damaged brain tissue and contralateral lateral cerebral tissue decreased markedly. Finally, the 3D brain reconstruction of rats showed that compared with the NS group, the brain injury volume of rats in the group administered higher‐dose LTKL was visibly reduced (Figure 3).

### 3.3 TUNEL staining

**Figure 4:**
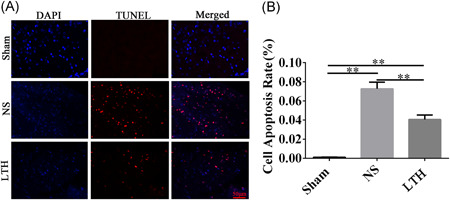
LTKL reduces the apoptosis of nerve cells after brain trauma. (A) The apoptosis of neurocyte was confirmed by TUNEL. Scale bar = 50 μm. (B) TUNEL was used to examine the number of apoptotic nerve cells at 1 day postoperation. The result is shown as mean ± SD. LTKL, Lu Tong Ke Li; TUNEL, terminal deoxynucleotidyl transferase deoxy‐UTP‐nick end labeling. **p < 0.01 [Color figure can be viewed at wileyonlinelibrary.com]

TUNEL staining on the first day after the operation was performed in each group. The apoptosis rate was higher in TBI rats than in the sham group. Compared with the NS group, the apoptosis rate of the LTH group was lower, with a statistically significant difference (Figure 4).

### 3.4 LTKL reduced inflammatory cytokine expression

**Figure 5:**
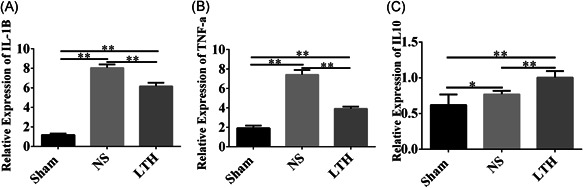
LTKL treatment reduces the level of inflammatory cytokines and increases the level of anti‐inflammatory cytokines after brain trauma. (A, B, C) RT‐PCR analysis was used to determine the mRNA level of IL‐1β, TNF‐a, and IL‐10 at 1 day after injury. The results are presented as mean ± SD. LTKL, Lu Tong Ke Li; IL‐1β, interleukin 1β; IL‐10, interleukin 10; mRNA, messenger RNA; RT‐PCR, reverse‐transcription polymerase chain reaction; TNF‐α, tumor necrosis factor‐α. *p < 0.05, **p < 0.01

The expression of inflammatory cytokines in the brain tissues of periencephalic injury was determined by RT‐PCR. IL‐1β, TNF‐α, and IL‐10 showed low expression in the sham group, while their expression was upregulated in the cortex of TBI. Treatment with LTKL clearly reduced the level of the pro‐inflammatory factors and increased the expression of IL‐10 (Figure 5).

## 4 DISCUSSION

In this study, we utilized LTKL for treatment of rats with TBI and found that LTKL significantly improved the neurological function damaged due to TBI. In addition, LTKL treatment significantly decreased the high‐signal region of the injured lateral brain tissue and the signal intensity in the injured site. In addition, administration of LTKL reduced the apoptosis rate in the injured lateral brain tissue. Finally, we examined the possible mechanism of LTKL treatment, and the results showed that LTKL alleviated the expression of inflammation cytokines. This evidence implied that treatment with LTKL may improve neurological function, and the mechanism is related to the regulation of inflammation.

### 4.1 LTKL treatment ameliorates damage to neurological function caused by TBI in rats

By TUNEL staining, the results showed that LTKL treatment produced fewer apoptotic cells in the lesion area. MRI was used to detect the brain injury in rats with TBI 10 days after surgery, and the results show that LTKL treatment led to reduced lesion volume compared with the NS control. These results implied that treatment with LTKL can attenuate the outcome of TBI associated with morphological improvement. Many studies have shown that apoptosis was closely related to brain trauma, and the apoptosis of nerve cells caused by brain trauma was a major type of cell death secondary to brain injury.^23^, ^24^ Some studies have been carried out using traditional Chinese medicine for the treatment of brain damage and find it resultful.^25^ TCM, derived from natural sources, has been widely applied for medical treatment for many years, and it has many advantages, such as low cost, easy accessibility, and fewer side effects.^26^, ^27^ Several TCMs, which were effective and safe, have been used for the treatment of TBI.^28^ Extracts of notopterygium, one of the components of LTKL, have been shown to have anti‐neuroinflammatory effects and could be used to treat Alzheimer's disease.^29^ Ligusticum, one of the components of LTKL, has been used to treat stroke for hundreds of years. Recent studies suggested that ligusticum can promote the production of neural differentiation factors and protect neurons.^30^ These results suggested that Cranial tong granule has the potential to repair nerve injury. The effective efficacy of LTKL makes it a reasonable choice for treating TBI.^31^

### 4.2 Exploration of the mechanism of LTKL in TBI rats

Subsequently, we explored the mechanism by which LTKL improves functions in TBI, and found that administration of LTKL obviously upregulated the protein level of IL‐10, and decreased the level of IL‐1β and TNF‐α in damaged brain tissue on the first day after TBI. In the literature, it has been shown that inflammation is a critical process in TBI events.^32^, ^33^ An increase in IL‐1β has been observed as early as 60 min after trauma.^34^ Within 24 h of injury, the expression levels of IL‐1β seem to be associated with the extent of injury.^35^ TNF, a vital pro‐inflammatory factor, is generated by microglia and astrocytes. Considerable evidence has shown that the upregulated expression of TNF is harmful.^36^ In the lesion area, increased TNF was expressed in damage area in advance of leukocyte cell.^37^ In addition, we found that IL‐10 was upregulated rapidly in brain tissue after injury. The literature reported IL ‐ 10 was increase at 4 h after injury, then maintained in a high level for at least 20 h, which showed an inhibition for others cytokine.^10^, ^38^ Therefore, inflammation may play an indispensable role in LTKL treating TBI.

## 5 CONCLUSION

On the basis of behavioral and morphological observations, this study demonstrated that LTKL can improve the neurobehavior of TBI injury. The neuroprotective effect was probably related to regulation of inflammation genes, such as upregulated expression of IL‐10, and downregulated expression of TNF‐α and IL‐1β. ‐This study provides crucial evidence of LTKL in the treatment of TBI, and provides a new treatment idea for future clinic practice.

## CONFLICTS OF INTEREST

The authors declare no conflicts of interest.

## ETHICS STATEMENT

The protocols of the experiment were authorized by the Institutional Medical Experimental Animal Care Committee of Kunming Medical University, China (No, 2021‐759).

## AUTHOR CONTRIBUTIONS

Jin Huang was involved in the conception of the study and revision of the paper. Ting‐Ting Li and Jie‐Chen were responsible for designing the study, and writing and revision of the manuscript. Ting‐Ting Li, Ji‐Lin Chen, and Rui‐Ze Niu were responsible for data description and submission of the manuscript. Jie‐Chen, Yong Chen, and Ting‐Bao Chen established the model, performed TUNEL staining, and harvested tissue. All authors have read and approved the final version of the manuscript.

## Data Availability

The analyzed data sets generated during the current study are available from the corresponding author on reasonable request.
